# Supplementary material for: Index of Cancer-Associated Fibroblasts Is Superior to the Epithelial–Mesenchymal Transition Score in Prognosis Prediction
Source: Cancers (Basel). 2020 Jun 28;12(7):1718. doi: 10.3390/cancers12071718 (PMC7408083; doi:10.3390/cancers12071718)
Supplement: Supplementary file 1 [file cancers-12-01718-s001.pdf]

# Supplementary Material: Index of Cancer-Associated Fibroblasts Is Superior to the Epithelial–Mesenchymal Transition Score in Prognosis Prediction

**Ying-Chieh Ko, Ting-Yu Lai, Shu-Ching Hsu , Fu-Hui Wang, Sheng-Yao Su, Yu-Lian Chen, Min-Lung Tsai, Chung-Chun Wu, Jenn-Ren Hsiao, Jang-Yang Chang, Yi-Mi Wu, Dan R. Robinson, Chung-Yen Lin and Su-Fang Lin**

## 1. Supplementary Figures

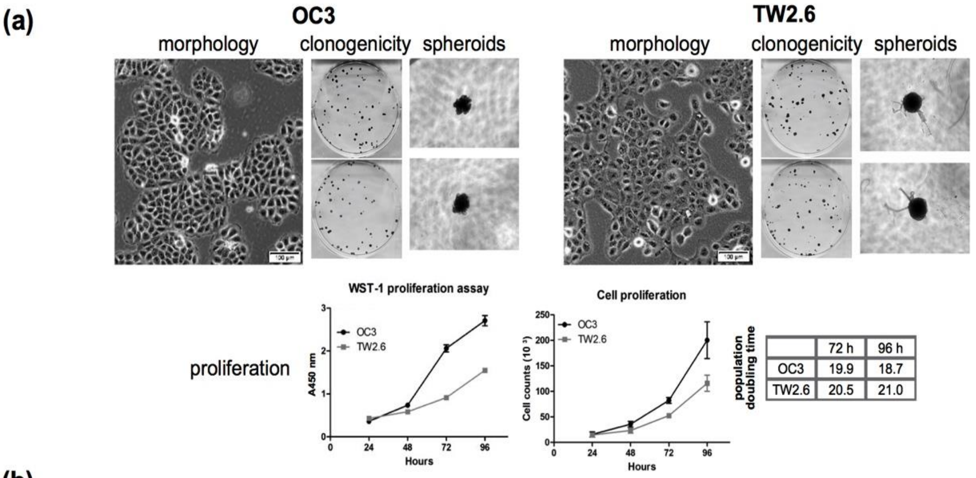


**(b)**

| | PartekFlow_HISAT2_XenoFilteR | | | | | Xenome | | | | | |
|---|---|---|---|---|---|---|---|---|---|---|---|
| Sample ID | total | human | mouse | Others | Sample ID | total | human | mouse | both | ambiguous | neither |
| OC3-NOG01 | 41,471,895 | 28,460,414 | 12,190,173 | 821,308 | OC3-NOG01 | 41,517,383 | 28,029,353 | 12,276,003 | 134,681 | 975,944 | 101,402 |
| OC3-NOG03 | 42,313,589 | 31,209,536 | 10,385,464 | 718,589 | OC3-NOG03 | 42,353,148 | 30,734,427 | 10,447,230 | 71,811 | 1,035,256 | 64,424 |
| OC3-NOG04 | 39,963,604 | 26,740,316 | 12,424,179 | 799,109 | OC3-NOG04 | 39,997,897 | 26,336,510 | 12,494,910 | 72,330 | 1,023,592 | 70,555 |
| OC3-NOG05 | 44,982,542 | 30,080,198 | 14,064,061 | 838,283 | OC3-NOG05 | 45,013,746 | 29,627,916 | 14,157,829 | 74,616 | 1,057,009 | 96,376 |
| OC3-NOG06 | 44,498,707 | 20,495,914 | 22,898,066 | 1,104,727 | OC3-NOG06 | 44,579,995 | 20,100,426 | 23,149,086 | 74,264 | 1,174,916 | 81,303 |
| OC3-NOG08 | 18,991,855 | 13,476,414 | 5,163,847 | 351,594 | OC3-NOG08 | 18,992,084 | 13,260,608 | 5,154,072 | 26,044 | 548,212 | 3,148 |
| TW2.6-NOG07 | 45,739,782 | 40,595,127 | 4,437,510 | 707,145 | TW2.6-NOG07 | 45,767,962 | 39,890,968 | 4,460,034 | 135,377 | 1,133,734 | 147,849 |
| TW2.6-NOG08 | 44,509,967 | 37,819,003 | 6,059,922 | 631,042 | TW2.6-NOG08 | 44,535,081 | 37,137,979 | 6,090,260 | 90,010 | 1,121,545 | 95,287 |
| TW2.6-NOG09 | 46,540,933 | 37,969,664 | 7,838,264 | 733,005 | TW2.6-NOG09 | 46,600,991 | 37,252,699 | 7,885,405 | 110,807 | 1,265,000 | 87,080 |
| TW2.6-NOG10 | 39,921,892 | 34,221,142 | 4,654,397 | 1,046,353 | TW2.6-NOG10 | 40,159,093 | 33,644,305 | 4,824,968 | 539,679 | 1,014,588 | 135,553 |
| TW2.6-NOG14 | 17,375,551 | 15,151,224 | 1,998,324 | 226,003 | TW2.6-NOG14 | 17,375,735 | 14,776,655 | 2,022,112 | 27,608 | 546,205 | 3,155 |

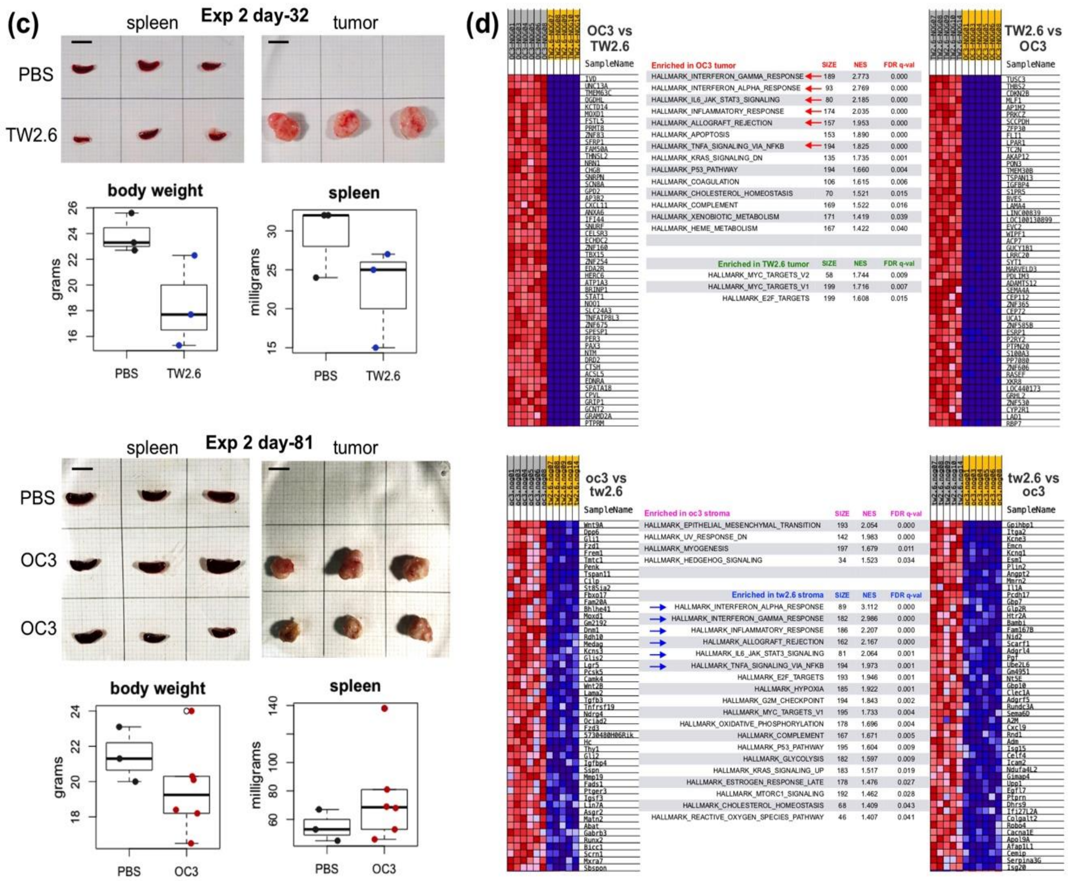


**Figure S1.** Supplementary information related to Figure 1. (**a**) Comparative analysis of morphology (scale bar = 100 µm), clonogenicity, spheroid formation, and proliferation between OC3 and TW2.6 cells grown in vitro; (**b**) Breakdown of RNA-seq reads generated from pipeline 1 (XenoFilteR) and pipeline 2 (Xenome); (**c**) Comparisons of spleens and tumors from control mice (PBS) with that from OC3-NOG (*n* = 6) and TW2.6-NOG (*n* = 3) in Exp 2. To reach ~500 mm$^3$ in tumor size, OC3-NOG were euthanized on day 81; TW2.6-NOG were euthanized on day 32. Scale bar, 1 cm. (**d**) Gene set enrichment analysis (GSEA) of tumor cell reads (upper) and murine stroma reads (lower). Heat maps show top 50 genes differentially expressed in OC3 tumor (upper left), TW2.6 tumor (upper right), OC3 stroma (lower left) and TW2.6 stroma (lower right). Expression values of high, moderate, low, and lowest are represented as red, pink, light blue, and dark blue, respectively [1]. Reports of enriched pathways for each analysis are listed in the middle. Pathways with FDR q-value > 0.05 were removed.

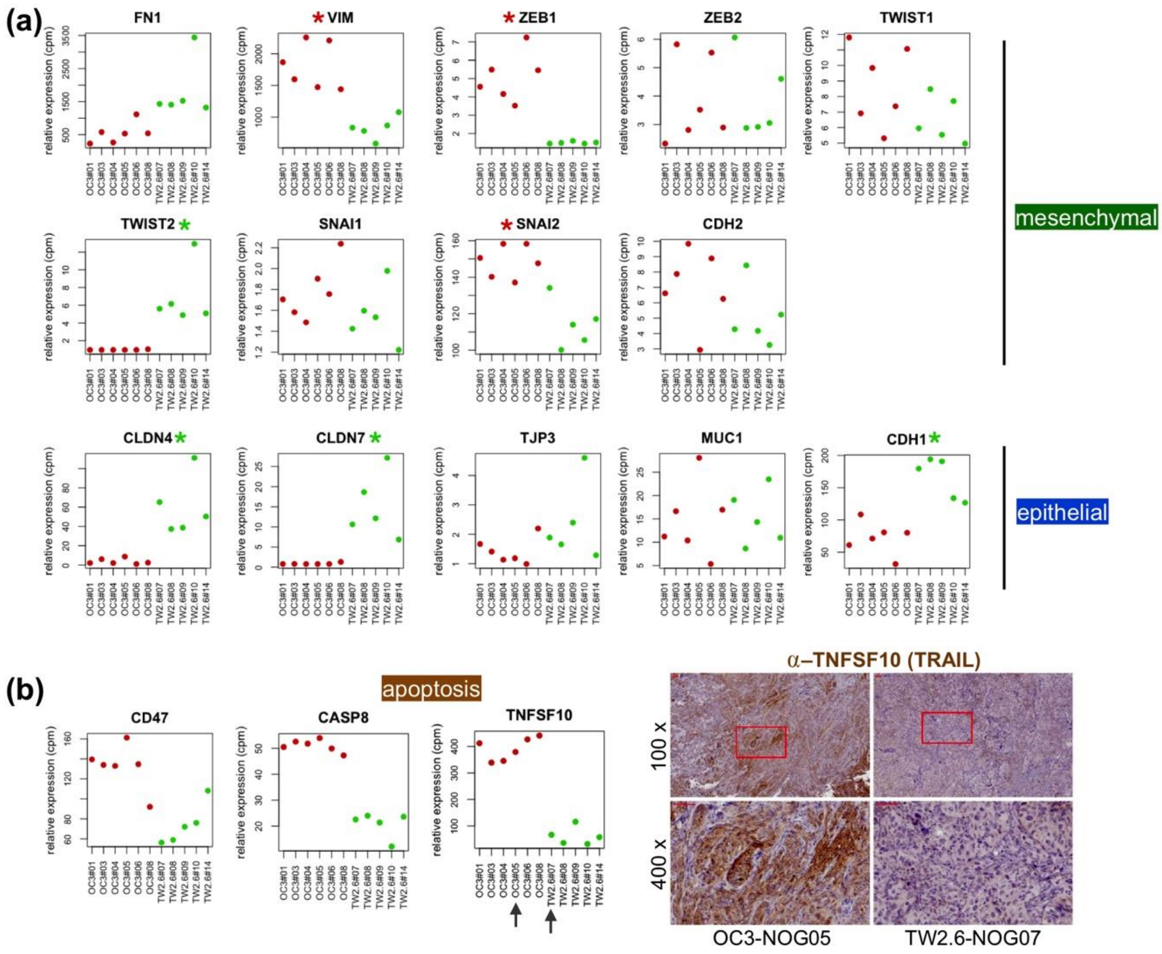


**Figure S2.** Supplementary information related to Figure 2. (**a**) Plots of 14 EMT related differentially expressed genes in OC3 and TW2.6 tumor cells, including mesenchymal genes (FN1, VIM, ZEB1, ZEB2, TWIST1, TWIST2, SNAI1, SNAI2, CDH2), and epithelial genes (CLDN4, CLDN7, TJP3, MUC1, CDH1); (**b**) Plots of apoptosis related DEGs (CD47, CASP8, TNFSF10) in tumor cells. Representative immunohistochemistry images of indicated xenograft sections stained for human TNFSF10 (brown). Scale bar, 50 μm.

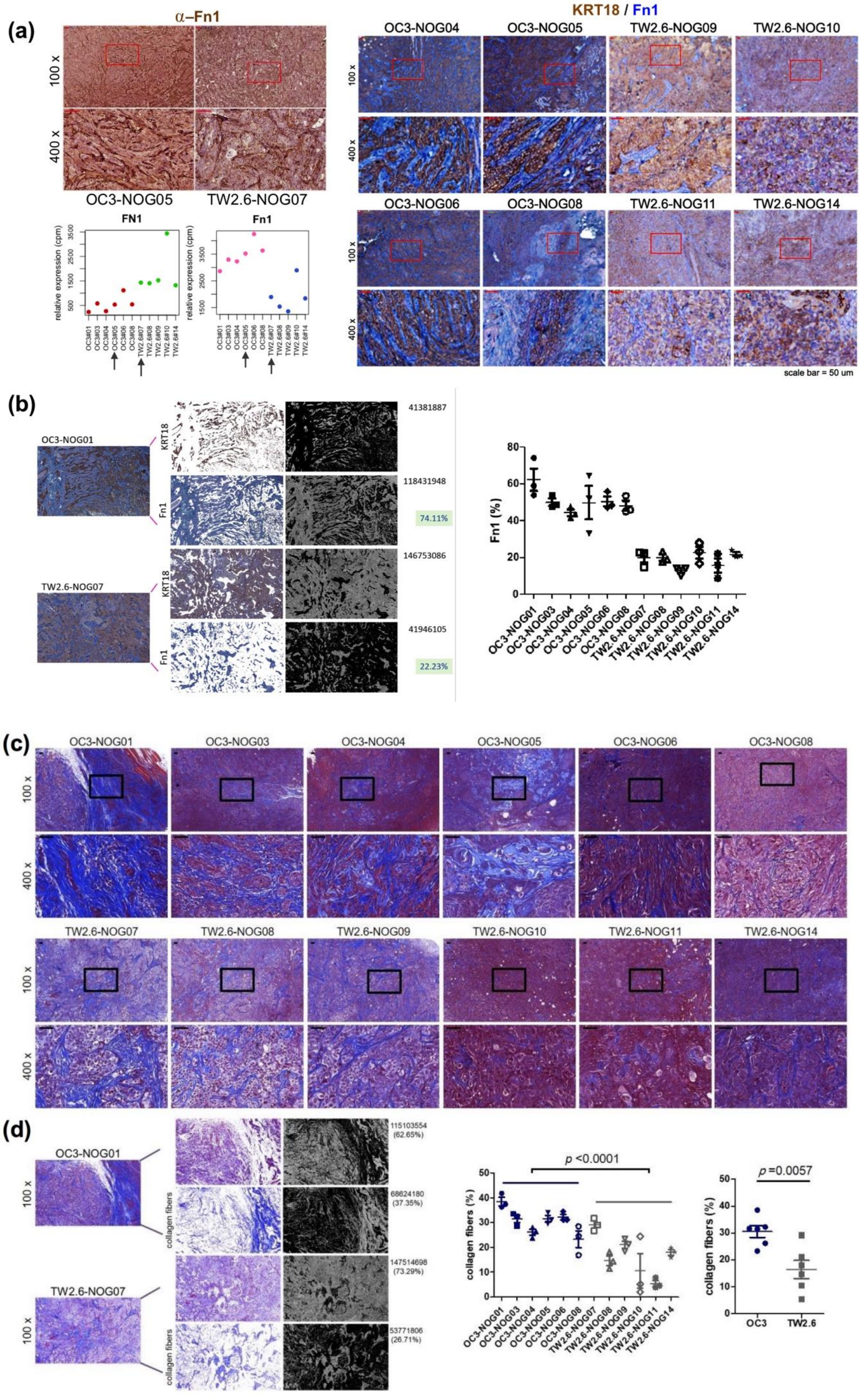
(a)
α–Fn1
100 x
400 x
OC3-NOG05
TW2.6-NOG07
FN1
Fn1
relative expression (cpm)
KRT18 / Fn1
OC3-NOG04
OC3-NOG05
TW2.6-NOG09
TW2.6-NOG10
OC3-NOG06
OC3-NOG08
TW2.6-NOG11
TW2.6-NOG14
scale bar = 50 um
(b)
OC3-NOG01
TW2.6-NOG07
KRT18
Fn1
41381887
118431948
74.11%
146753086
41946105
22.23%
Fn1 (%)
(c)
OC3-NOG01
OC3-NOG03
OC3-NOG04
OC3-NOG05
OC3-NOG06
OC3-NOG08
TW2.6-NOG07
TW2.6-NOG08
TW2.6-NOG09
TW2.6-NOG10
TW2.6-NOG11
TW2.6-NOG14
(d)
OC3-NOG01
TW2.6-NOG07
collagen fibers
115103554 (62.65%)
68624180 (37.35%)
147514698 (73.29%)
53771806 (26.71%)
p <0.0001
collagen fibers (%)
p =0.0057
OC3
TW2.6

**Figure S3.** Supplementary information related to Figure 3. (**a**) (left) Representative immunohistochemistry images of indicated tissue sections stained for murine Fn1 (brown). Scale bars, 50 µm. Plots denote expression levels (cpm) of human FN1 and murine Fn1 in OC3 and TW2.6 CDXs. Arrows indicate samples used for IHC staining experiments. (right) Representative IHC images of indicative sections co-stained with human KRT18 (brown) and murine Fn1 (blue). (**b**) Quantitative analysis of dual IHC staining depicted in (**a**). Note that TW2.6-NOG11 was not included in RNA-seq analysis. (**c**) Representative Masson's trichrome stain of OC3 and TW2.6 sections (collagen: blue; cytoplasm: pink; nuclei: dark brown). Scale bar, 50 µm. (**d**) Quantification of Masson's trichrome stain depicted in (**c**). Results show mean ± SEM of three 100x magnification fields from each section.

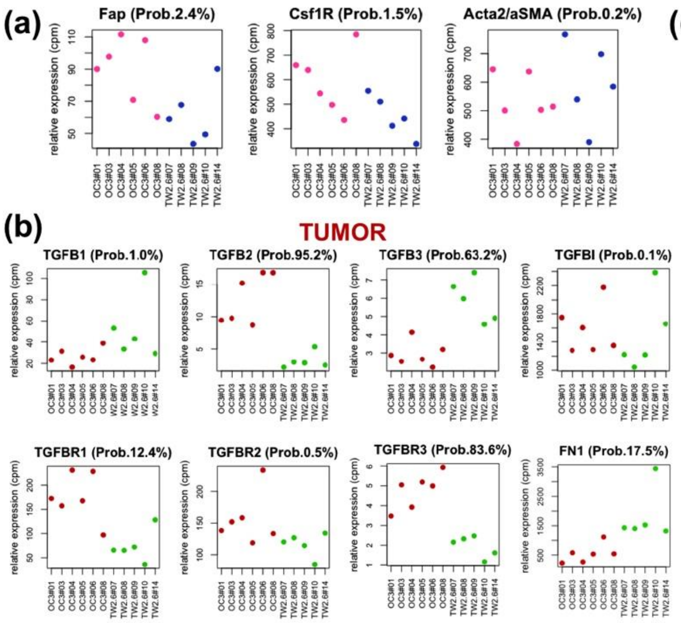


(c)

| Covariate | HR (95% CI for HR) | p.value |
|---|---|---|
| LNmeta | 1.8 (0.65-5) | 0.26 |
| AngioLymphatic invasion (ALI) | 1.9 (0.67-5.3) | 0.23 |
| **EMT_Score** | **4.2 (1.3-14)** | **0.015*** |
| TGFB1 | 0.65 (0.23-1.8) | 0.41 |
| **TGFB2** | **4.9 (1.4-17)** | **0.015*** |
| TGFB3 | 0.89 (0.32-2.5) | 0.83 |
| **TGFBI** | **11 (2.4-50)** | **0.0021**** |
| TGFBR1 | 1.4 (0.49-3.8) | 0.55 |
| TGFBR2 | 1.3 (0.49-3.7) | 0.57 |
| TGFBR3 | 0.56 (0.2-1.6) | 0.27 |
| FN1 | 1.8 (0.63-5) | 0.28 |

NCKU-OrCA-40T (GSE37991)

**Figure S4.** Supplementary information related to Figure 4. (**a**) Plots denote expression of genes conventionally attributed to activated fibroblasts (Fap, Csf1R) and myoblasts (Acta2) in the stroma of OC3 and TW2.6 CDXs. (**b**) Plots denote RNA-seq reads of human TGF-β related genes and FN1 in OC3 and TW2.6 CDXs. (**c**) Cox proportional hazard ratios of two clinical features (LN meta and angiolymphatic invasion), EMT score, indicated TGF beta-axis genes, and FN1 for the 40 OSCC patients in the NCKU-OrCA-40TN cohort (GSE37991).

## 2. Supplementary Materials and Methods

### *Text S1: Clonogenic Survival Assay*

Cells were cultured in six-well plates (100 cells per well) for 10 to 14 d. Cells were fixed with 70% ethanol for 30 min at 4 °C, stained with 1% crystal violet for 30 min, and rinsed with water thoroughly. The stained colonies (>50 cells) were counted under a microscope. Colony count for each treatment was presented as mean ± SD derived from quadruple wells.

### *Text S2: Spheroid Invasion Assays*

The Cultrex 96 Well 3D Spheroid BME Cell Invasion Assay kit (#3500-096-K; Trevigen, Gaithersburg, MD, USA) was used according to the manufacturer's protocol. In each assay, 1,500 OC3 or TW2.6 cells resuspended in spheroid formation solution were seeded in a 96-well round bottom plate to form aggregated spheroids (72 h), followed by adding invasion matrix (72 h). The bright-field spheroids images were captured by using an Olympus IX73 microscope.

### *Text S3: Cell Proliferation Assays*

Cell proliferation was determined by using a WST-1 assay (Roche, Indianapolis, IN, USA) or trypan blue exclusion method (Figure S1a). In WST-1 assay, 2000 cells per well were seeded in a 96-well plate and WST-1 proliferation assay was performed on the following 4 consecutive days. 10 μL of WST-1 reagent was added to each well containing 100 μL of medium. Four hours after incubation at 37 °C, the microplate was gently mixed for 1 min and the absorbance was measured by microplate reader Spectramax 250 (Molecular Devices, Hampton, NH, USA) at 450 nm with a reference wavelength at 630 nm. In parallel, for each sample at each time point, 5000 cells per well were seeded in a 48-well plate, followed by trypan blue exclusion assay to determine cell viability.

## Reference

1. Subramanian, A.; Tamayo, P.; Mootha, V.K.; Mukherjee, S.; Ebert, B.L.; Gillette, M.A.; Paulovich, A.; Pomeroy, S.L.; Golub, T.R.; Lander, E.S.; et al. Gene set enrichment analysis: A knowledge-based approach for interpreting genome-wide expression profiles. *Proc. Natl. Acad. Sci.* **2005**, *102*, 15545–15550, doi:10.1073/pnas.0506580102.

© 2020 by the authors. Licensee MDPI, Basel, Switzerland. This article is an open access article distributed under the terms and conditions of the Creative Commons Attribution (CC BY) license (http://creativecommons.org/licenses/by/4.0/).
